# Validity of Diagnostic Algorithms for Inflammatory Bowel Disease in Japanese Hospital Claims Data

**DOI:** 10.3390/ijerph19137933

**Published:** 2022-06-28

**Authors:** Sayumi Takahashi, Taku Obara, Yoichi Kakuta, Yusuke Shimoyama, Takeo Naito, Rintaro Moroi, Masatake Kuroha, Hisashi Shiga, Yoshitaka Kinouchi, Atsushi Masamune

**Affiliations:** 1 Division of Gastroenterology, Tohoku University Graduate School of Medicine, 1-1 Seiryo, Aoba, Sendai 980-8574, Japan; sayumi.takahashi.d4@med.tohoku.ac.jp (S.T.); low.life41@gmail.com (Y.S.); takeo.naito24@gmail.com (T.N.); rinta@med.tohoku.ac.jp (R.M.); kuroha@med.tohoku.ac.jp (M.K.); shiga@med.tohoku.ac.jp (H.S.); amasamune@med.tohoku.ac.jp (A.M.); 2 Division of Preventive Medicine and Epidemiology, Tohoku Medical Megabank Organization, Tohoku University, Sendai 980-8573, Japan; obara-t@hosp.tohoku.ac.jp; 3 Department of Pharmaceutical Sciences, Tohoku University Hospital, Sendai 980-8574, Japan; 4 Student Healthcare Center, Institute for Excellence in Higher Education, Tohoku University, Sendai 980-8576, Japan; ykinouchi@med.tohoku.ac.jp

**Keywords:** validity, inflammatory bowel disease, claims database, diagnostic algorithm

## Abstract

Inflammatory bowel disease (IBD) diagnoses are increasing in Japan. Some patients have symptoms that are difficult to control, and further research on IBD is needed. Claims databases, which have a large sample size, can be useful for IBD research. However, it is unclear whether the International Classification of Diseases, Tenth Revision (ICD-10) codes alone can correctly identify IBD. We aimed to develop algorithms to identify IBD in claims databases. We used claims data from the Department of Gastroenterology, Tohoku University Hospital from 1 January 2016 to 31 December 2020. We developed 11 algorithms by combining the ICD-10 code, prescription drug, and workup information. We had access to the database which contains all the information for Crohn’s disease and ulcerative colitis patients who visited our department, and we used it as the gold standard. We calculated sensitivity, specificity, positive predictive value (PPV), and negative predictive value for each algorithm. We enrolled 19,384 patients, and among them, 1012 IBD patients were identified in the gold standard database. Among 11 algorithms, Algorithm 4 (ICD-10 code and ≥1 prescription drugs) showed a strong performance (PPV, 94.8%; sensitivity, 75.6%). The combination of an ICD-10 code and prescription drugs may be useful for identifying IBD among claims data.

## 1. Introduction

Inflammatory bowel disease (IBD) is a general term for Crohn’s disease (CD) and ulcerative colitis (UC) [1]. IBD causes digestive disorders and inflammation in the gastrointestinal tract, and although various treatments are currently available [1], some patients have symptoms that are difficult to control.

The number of IBD patients is increasing worldwide, including in Japan [2]. While IBD is most commonly diagnosed in young people, the number of elderly patients with ulcerative colitis has been increasing in Japan in recent years [3]. In addition, it is known that Japanese patients with Crohn’s disease are more often diagnosed amongst male patients than those in Western countries [4]. A large country-wide survey is needed to more accurately characterize these epidemiological features.

In addition, side-effects of drugs for IBD have been reported, including mesalamine intolerance [5,6,7], allergic reactions to biological medicine [8], and thiopurine-induced leukopenia and alopecia [9,10]. For thiopurine, side effects can be avoided by examining NUDT15 codon 139 [9,11,12], but there is still no clear way to avoid side effects with other drugs. Therefore, further research on IBD is needed to provide optimal treatment for each patient and to minimize side effects. However, although the number of IBD patients is increasing, there are few cases at a single center.

Claims databases consist of billing codes submitted to payers by physicians, pharmacies, hospitals, and other health care providers and include information such as the names of diseases, procedures, and medications [13], and various claims databases are available in Japan [14]. One advantage of claims databases is that they have a large sample size [15], so claims databases can be useful for research on IBD. Some studies have used claims databases for IBD research [16,17]. Claims databases are particularly useful in conducting studies on patients with special situations, for which single-center studies do not have enough cases. For example, cases with rare complications or rare side effects require large datasets. Since IBD has a highest incidence among 20- to 29-year-olds [18], pregnancy and childbirth are important life events for female IBD patients. The safety of drugs for pregnant women is also an issue, and there are some reports using claims databases to assess pregnancy outcomes and their effects on the fetus [19,20]. Thus, claims databases can also be useful for assessing the effects of drugs on pregnant women with IBD.

However, when using claims databases, it is unclear whether International Classification of Diseases, Tenth Revision (ICD-10) codes alone can correctly identify IBD [21], and it is necessary to assess the validity of diagnostic algorithms. Therefore, we aimed to develop algorithms to identify IBD in the claims databases.

## 2. Methods

### 2.1. Study Population and Algorithm Development

This study used claims data from all 19,452 outpatients and inpatients at the Department of Gastroenterology, Tohoku University Hospital, from 1 January 2016 to 31 December 2020. The claims data included the identification number, ICD-10 codes, birth date, sex, visit date, prescription drugs, workups, and procedures. We developed 11 algorithms to identify IBD by combining the ICD-10 codes (CD: K-50, UC: K-51), prescription drugs, and workups (Table 1). Prescription drugs included the following: oral 5-aminosalicylates (mesalazine and salazosulfapyridine), topical medication (5-aminosalicylates and steroids), thiopurine (azathioprine and mercaptopurine), biological medicine (infliximab, adalimumab, golimumab, vedolizumab, and ustekinumab), zentacoart, and elemental diet. Workups included the following: colonoscopy, small bowel series, and capsule endoscopy.

Prescription drugs and workups were determined in accordance with the Japanese guidelines for IBD [22]. Oral or injectable steroids and tacrolimus were used for IBD and other diseases; thus, we did not include them with the prescription drugs.

### 2.2. IBD Gold Standard

In the Lower Gastrointestinal Disease group at the Department of Gastroenterology, Tohoku University Hospital, we created and continue to manage a database that includes all patients with small intestine or colon diseases, except for neoplastic disease. The database includes the following diseases: CD, UC, IBD unclassified, familial Mediterranean fever, intestinal Behcet’s disease, mesenteric panniculitis, non-specific multiple ulcers of the small intestine, simple ulcer, and gastric amyloidosis. We update the patients’ consultation day and summary information in the database each time they visit the Department of Gastroenterology.

Because the database has been in operation since 2010 and it includes patients between January 2016 and December 2020, we used it as the gold standard in this study.

### 2.3. Statistical Analysis

We calculated the sensitivity, specificity, positive predictive value (PPV), and negative predictive value (NPV) for each algorithm compared with the CD and UC database in the Department of Gastroenterology. A subgroup analysis was conducted to determine whether the ICD-10 code classification was appropriate as a method of classifying CD and UC for IBD, which was identified using the appropriate algorithm. We also calculated the sensitivity, specificity, PPV, and NPV. All statistical analyses were performed using SAS version 9.4 (SAS Institute Inc., Cary, NC, USA).

## 3. Results

### 3.1. Patient Characteristics

In this study, we first extracted data from 19,452 patients. We excluded patients with a suspected ICD-10 code only, and 19,384 patients were enrolled. Among these patients, 1012 IBD patients were identified using the gold standard (Table 2). Among the 1012 IBD patients, 507 had CD, and 505 had UC. The mean age of the patients was 43.8 ± 15.3 years, and there were 646 men (63.8%) and 366 women (36.2%). The most prescribed drug was oral 5-aminosalicylates (*n* = 709, 70.1%). Among the biological medications, the most prescribed was infliximab (*n* = 190, 18.8%) and adalimumab (*n* = 190, 18.8%).

### 3.2. Algorithm Performance

The IBD algorithm performance is shown in Table 3. All algorithms had a high specificity and NPV (≥95%). Algorithm 7 (96.8%) showed the highest PPV, followed by Algorithm 4 (94.8%), Algorithm 5 (94.8%), Algorithm 6 (90.8%), and Algorithm 1 (84.2%). Algorithm 3 (53.2%) showed the lowest PPV, followed by Algorithm 11 (55.5%), Algorithm 10 (57.1%), Algorithm 8 (64.3%), Algorithm 2 (67.4%), and Algorithm 9 (67.7%). Algorithm 11 (96.9%) showed the highest sensitivity, followed by Algorithm 8 (96.4%), Algorithm 9 (89.1%), Algorithm 10 (88.3%), Algorithm 1 (86.4%), Algorithm 2 (85.7%), and Algorithm 4 (75.6%). Algorithm 7 (26.9%) showed the lowest sensitivity, followed by Algorithm 5 (29.1%), Algorithm 6 (29.2%), and Algorithm 3 (31.8%). Among all algorithms, Algorithm 4 had the highest PPV and sensitivity scores. Therefore, it was suggested that Algorithm 4 was a valid algorithm.

### 3.3. Subgroup Analyses

Using data from IBD patients identified by Algorithm 4, which was a method of identifying CD and UC patients, we calculated the sensitivity, specificity, PPV, and NPV of CD and UC using the corresponding ICD-10 codes (K50 and K51, respectively). Both the K50 and K51 ICD-10 codes had a high sensitivity, specificity, PPV, and NPV (Table 4).

## 4. Discussion

In this study, we developed 11 algorithms using a combination of the ICD-10 codes, prescription drugs, and workups. Algorithm 1, which included only the ICD-10 code, had a high PPV of 84.2%, but in combination with prescription drugs and workups, the PPV was even higher. Algorithm 7 (one ICD-10 code, ≥1 prescription drug, and ≥1 workup) had the highest PPV (96.8%) but the lowest sensitivity (26.9%). Because IBD is not a high-prevalence disease, adapting an algorithm with a low sensitivity makes it difficult to identify a sufficient number of patients in claims databases. Algorithm 5 (one ICD-10 code and ≥1 workup), which had a higher PPV (94.8%), also had a low sensitivity (29.1%). Therefore, we determined that Algorithm 4 (one ICD-10 code and ≥1 prescription drug), which had a higher PPV (94.8%) and sensitivity (75.6%) than those of the other algorithms, was the appropriate algorithm. In subgroup analyses, both CD and UC classifications using the ICD-10 code had a high PPV, sensitivity, specificity, and NPV for IBD, which was identified by Algorithm 4. The ICD-10 code was useful for classifying patients as CD or UC from the IBD patients who were identified using Algorithm 4.

Previous studies have developed some diagnostic algorithms for IBD. Lee et al. [23] reported that a combination of the ICD-10 code, ≥1 health care encounter, and ≥1 pharmaceutical prescription for IBD-specific drugs achieved excellent performance in identifying patients with IBD (sensitivity, 93.1%; specificity, 98.1%; PPV, 97.5%; NPV, 98%). In our study, the participants were all outpatients or inpatients at the Department of Gastroenterology, Tohoku University Hospital, and the best algorithm was a combination of the ICD-10 code and ≥1 prescription drug for IBD, which was a result similar to that in the above study. Lee et al. defined IBD-specific medications as oral or topical 5-aminosalicylates, thiopurine, and biological medicine (infliximab, adalimumab). We added topical steroids, zentacoart, and elemental diet to IBD-specific medications, as defined by Lee et al. We also added golimumab, vedolizumab, and ustekinumab as biologic medicines. All of these drugs are commonly used in the current treatment of IBD, and the Lee et al.’s study may have missed patients using them. In any case, this study is important because the treatment of IBD and the drugs indicated for it vary from country to country, even if the results are similar to previous studies. We examined the ICD-10 codes, prescription drugs, and medical workup results. Workups alone had a low PPV, but when combined with the ICD-10 code and the prescription, such as in algorithms 5, 6, and 7, there was a high PPV. The low sensitivity of algorithms 5, 6, and 7 may be because some of the patients had already undergone workups at their hospital of origin and had not yet undergone workups in our department.

Claims databases have large sample sizes, and they are useful for performing research on data from a small number of patients. However, the validity of diagnostic algorithms for secondary use needs to be assessed. In Japan, a validation study has been conducted for CD [24], but no validation studies have been conducted for IBD. Thus, this study will be useful for future research on IBD using claims databases in Japan.

The present study has some limitations. First, the Department of Gastroenterology at Tohoku University Hospital has a high level of expertise in IBD, and the subjects of this study were all patients who were seen or admitted there, which may have increased the PPV. Second, this study included only patients who visited the Department of Gastroenterology and not patients who visited other departments. This study could not include UC patients after total colectomy who only visited the Department of Gastrointestinal Surgery, because they did not require medications or workups. Third, because this study was conducted at a single center, we do not know if it can be adapted to other centers.

## 5. Conclusions

We developed an algorithm to identify IBD using claims data at the Department of Gastroenterology. We are planning to apply this algorithm to other claims databases in Japan in future research.

## Figures and Tables

**Table 1:** IBD algorithms.

No.	Algorithm
1	ICD-10 code
2	≥1 prescription drug
3	≥1 workup
4	ICD-10 code and ≥1 prescription drug
5	ICD-10 code and ≥1 workup
6	≥1 prescription drug and ≥1 workup
7	ICD-10 code and ≥1 prescription drug and ≥1 workup
8	ICD-10 code or ≥1 prescription drug
9	ICD-10 code or ≥1 workup
10	≥1 prescription drug or ≥1 workup
11	ICD-10 code or ≥1 prescription drug or ≥1 workup

IBD, Inflammatory bowel disease; ICD, International Classification of Diseases, Tenth Revision.

**Table 2:** Characteristics of IBD patients at Tohoku University hospital who were identified in the database.

Characteristic	No.
IBD	1012
CD	507
UC	505
Age (years, mean ± SD)	43.8 (±15.3)
Men/women (%)	646 (63.8)/366 (36.2)
Treatment (%)	
Oral 5-aminosalicylates	709 (70.1)
Topical medication	236 (23.3)
Thiopurine	308 (30.4)
Infliximab	190 (18.8)
Adalimumab	190 (18.8)
Golimumab	17 (1.68)
Vedolizumab	53 (5.24)
Ustekinumab	93 (9.19)
Zentacoart	45 (4.45)
Elemental diet	249 (24.6)

IBD, Inflammatory bowel disease; CD, Crohn’s disease; UC, ulcerative colitis; SD, standard deviation.

**Table 3:** IBD algorithm performance.

Algorithm	Identified Patients (*n*)	Sensitivity (%)	Specificity (%)	PPV (%)	NPV (%)
1	1038	86.4	99.1	84.2	99.3
2	1286	85.7	97.7	67.4	99.2
3	605	31.8	98.5	53.2	96.3
4	807	75.6	99.8	94.8	98.7
5	310	29.1	99.9	94.8	96.2
6	325	29.2	99.8	90.8	96.2
7	281	26.9	99.9	96.8	96.1
8	1517	96.4	97.1	64.3	99.8
9	1333	89.1	97.7	67.7	99.4
10	1566	88.3	96.3	57.1	99.3
11	1768	96.9	95.7	55.5	99.8

IBD, Inflammatory bowel disease; PPV, positive predictive value; NPV, negative predictive value.

**Table 4:** ICD-10 code performance using algorithm 4 to identify CD and UC patients in the subgroup analyses.

	No.	Sensitivity (%)	Specificity (%)	PPV (%)	NPV (%)
CD (ICD-10 code; K50)	427	98.6	95.2	95.6	98.4
UC (ICD-10 code; K51)	380	98.9	92.8	91.3	99.1

CD, Crohn’s disease; UC, ulcerative colitis; PPV, positive predictive value; NPV, negative predictive value; ICD, International Classification of Diseases, Tenth Revision.

## Data Availability

The data presented in this study are available on request from the corresponding author. The data are not publicly available due to privacy and ethical restrictions.
